# The Importance of Movement Velocity as a Measure to Control Resistance Training Intensity

**DOI:** 10.2478/v10078-011-0053-6

**Published:** 2011-10-04

**Authors:** Juan J. González-Badillo, Mário C. Marques, Luis Sánchez-Medina

**Affiliations:** 1 University Pablo de Olavide, Seville, Spain; 2 Department of Sport Sciences, University of Beira Interior (UBI), Covilhã, Portugal; 3 Research Centre for Sport Sciences, Health and Human Development (CIDESD), Vila Real, Portugal; 4 Studies, Research and Sport Medicine Center, Government of Navarra, Navarra, Spain

**Keywords:** velocity, strength, power, level of effort, testosterone, lactate

## Abstract

Configuration of the exercise stimulus in resitance training has been traditionally associated with a combination of the so-called ‘acute resistance exercise variables’ (exercise type and order, loading, number of repetitions and sets, rests duration and movement velocity). During typical resistance exercise in isoinertial conditions, and assuming every repetition is performed with maximal voluntary effort, velocity unintentionally declines as fatigue develops. However, few studies analyzing the response to different resitance training schemes have described changes in repetition velocity or power. It thus seems necessary to conduct more research using models of fatigue that analyze the reduction in mechanical variables such as force, velocity and power output over repeated dynamic contractions in actual training or competition settings. Thus, the aim of this paper was to discuss the importance of movement velocity concerning control training intensity.

## Introduction

If we are to speak about the design and implementation of resistance training programs, the first thing we must define is the very first term ‘training program’. A training program is the expression of an ordered sequence or series of efforts that have a dependency relationship to each other.

Since we have used the term ‘effort’ we must move ahead to define it. The meaning of this term must be understood in the sense of the actual degree of demand in relation to the current possibilities of a given subject. We call this ‘level of effort (LE)’ (González Badillo and Gorostiaga, 1993, 1995). Therefore, when we talk about strength or resistance training, the nature of the effort will be best defined by the number of repetitions actually performed in each exercise set with respect to the maximum possible number of repetitions that can be completed against a given absolute load. It thus seems reasonable that the degree or level of effort is substantially different when performing, e.g., eight out of twelve possible repetitions with a given load [8(12)] compared to performing all repetitions [12(12)].

Configuration of the exercise stimulus in resistance training mainly depends on the manipulation of three variables: type of exercise, volume and intensity. Once the exercises have been selected, the training load will be defined by the manipulation of volume and intensity. Of these two, the latter is the most important since it is the intensity which determines the amount of volume (number of repetitions) that can be performed. Furthermore, exercise intensity is generally acknowledged as the most important stimulus related to changes in strength levels. It is for these reasons that we will focus on the study of training intensity in the following paragraphs.

Exercise intensity during resistance training has been commonly identified with relative load (percentage of one-repetition maximum, 1RM) or with performing a given maximal number of repetitions in each set (XRM: 5RM, 10RM, 15 RM, etc.). However, for several reasons, none of these methods is entirely appropriate for precisely monitoring the real effort the athlete is performing in each training session.

The first approach requires coaches to individually assess the 1RM value for each athlete. It is true that expressing intensity as a percentage of the maximum repetition has the advantage that it can be used to program resistance training for many diferent athletes at the same time, the loads being later transformed in absolute values (kg) for each person. Another advantage is that this expression of the intensity can clearly reflect the dynamics of the evolution of the training load if we understand the percentage of 1RM as an effort, and not as a simple arithmetic calculus. This would yield valuable information about the type of training being prescribed. Direct assessment of 1RM, however, has some potential disadvantages worth noting. It may be associated with injury when performed incorrectly or by novice subjects and it is time-consuming and impractical for large groups. Furthermore, experience tells us that the actual RM can change quite rapidly after only a few training sessions and often the obtained value is not the subject’s true maximum.

An alternative way to prescribe loading intensity is to determine, through trial and error, the maximum number of repetitions that can be performed with a given submaximal weight. For example, 10RM refers to a weight that can be lifted ten times, but no more. Several studies have been conducted to identify the relationship between selected percentages of 1RM and the number of repetitions to failure, establishing a repetition maximum continuum. It is believed that certain performance characteristics are best trained using specific RM load ranges. This method certainly eliminates the need for a direct 1RM test, but it is not without drawbacks either. Although training using exhaustive efforts is common practice in strength training, increasing evidence (Sanborn et al., 2000; Folland et al., 2002; Izquierdo et al., 2006; Drinkwater et al., 2007) shows that training to repetition failure does not necessarily improve the magnitude of strength gains and that it may even be counterproductive by inducing excessive fatigue, mechanical and metabolic strain for subsequent sessions as well as undesirable transitions to slower fibre types (Fry, 2004). Fatigue associated with training to failure not only significantly reduces the force that a muscle can generate, but also the nervous system’s ability to voluntarily activate the muscles (Häkkinen, 1993). This could have adverse effects on rapid force production (‘rate of force development’, RFD), movement velocity and power of the vast majority of sports movements (Hakkinen and Kauhanen, 1989). Furthermore, after performing the first set to failure the number of repetitions in following sets is reduced, regardless of recovery. Hence, by the second or third set it is likely that the athlete may not be training within the prescribed intensity range. Therefore, this system, besides being very tiring and having shown no advantage over other lower effort types of training, it is unrealistic because it is practically impossible to know exactly how many repetitions can be done with a given absolute load without any initial reference. Furthermore, if in the first set the subject has completed the maximum number of repetitions, it will be very difficult –if not impossible– to perform the same number of reps in the following sets.

The aforementioned limitations suggest trying to find better ways to objectively monitor training load during resistance exercise. Movement velocity is another variable which could be of great interest for monitoring exercise intensity but surprisingly it has been vaguely mentioned in most studies to date. The lack of use of this variable is likely because until recently it was not possible to accurately measure velocity in typical isoinertial resistance training exercises. Thus, most of the research which has addressed velocity of movement in strength training has done so mainly in studies that used isokinetic dynamometry which, unfortunately, is not an ideal or common training setting. The actual velocity performed in each repetition could perhaps be the best reference to gauge the real effort which is being incurred by the athlete. This can be achieved with a well-measured level of effort (LE) in what we term ‘velocity-based resistance training’, a new and much more accurate and rational training paradigm.

Monitoring repetition velocity during resistance exercise seems important since both the neuromuscular demands and the training effect itself largely depend on the velocity at which loads are lifted. . The higher the velocity achieved against a given (absolute) load, the greater the intensity, and this will influence the training effect (González Badillo and Ribas, 2002).Thus, movement velocity is a key ingredient of training intensity. With this training approach, instead of a certain amount of weight to be lifted, coaches should prescribe resistance exercise in terms of two variables: 1) first repetition’s mean velocity, which is intrinsically related to loading intensity; and 2) a maximum percent velocity loss to be allowed in each set. When this percent loss limit is exceed the set must be terminated. The limit of repetition velocity loss should be set beforehand depending on the primary training goal being pursued, the particular exercise to be performed as well as the training experience and performance level of the athlete.

The importance that monitoring execution velocity in training exercise could provide for resistance traning programming was already noticed in 1991 (González-Badillo, 1991, p. 172). We have recently studied this hypothesis and we have been able to confirm some important practical applications that movement velocity provides as a determinant of the level of effort during resistance training as well as an indicator of the degree of fatigue (González-Badillo and Sánchez-Medina, 2010; Sánchez-Medina and González-Badillo, 2011).

The monitoring or control of movement velocity during training complements and refines the concept of ‘level of effort’ (LE), published by us in 1992 in the text “Methodology of strength training” (Master COE, Spanish Olympic Committee) since it truly represents a breakthrough in determining the degree or level of effort during resistance training. The LE not only takes into account the number of performed repetitions per set, but also the maximum number of repetitions that could be completed within the set, and is expressed by the ratio between the number of repetitions performed and those possible or achievable reps. Therefore, the nature of the effort is or expresses the very intensity and degree of loading, and is determined by two indicators: 1) the numerical difference between the number of performed repetitions and the maximal possible number; and 2) by the maximal number of repetitions you can perform within the set.

In a recent study (González-Badillo and Sánchez-Medina. Movement Velocity as a Measure of Loading Intensity in Resistance Training. Int J Sports Med 31: 347–352, 2010) the following conclusions were obtained:

▪ Each percentage of 1RM has its own mean velocity. This means that mean velocity attained in the first repetition within a set determines the real intensity of effort being incurred.
▪ Mean velocity attained with each percentage of 1RM remains stable after a subject’s RM value is modified following a period of strength training.
▪ Mean velocity attained with the 1RM (V1RM) determines the subtle changes that could take place in mean propulsive velocity (MPV) with each percentage of 1RM when a test is repeated after a training period.
▪ Only those repetitions whose mean concentric velocity is not greater that 0.20 m/s should be considered as true maximum repetitions. As V_1RM_ exceeds this figure, mean velocities attained with each % 1RM and relative loads themselves would deviate from their true values. This means that when V1RM is not actually measured, as frequently occurs, the values of mean velocity correspondent to each %1RM, as well as these percentages themselves, can easily differ from the true values.
▪ Movement velocity, expressed as mean propulsive velocity (MPV), can be considered as the steadiest variable for muscle strength assessment in isoinertial conditions.

In another study, we examined the acute physiological and mechanical responses to fifteen types of resistance training protocols performed with different level of effort (LE). Part of the results have already been published (Sánchez-Medina and González-Badillo. Velocity loss as an indicator of neuromuscular fatigue during resistance training. Med Sci Sports Exerc 2011; published ahead of print. DOI: 10.1249/MSS.0b013e318213f880). The main conclusions were:

▪ Relative reductions in: 1) Mean Propulsive Velocity (MPV) within a set, 2) MPV attained with the load that elicits a velocity of ∼1 m/s in resting conditions, and 3) vertical jump (CMJ) height, all can be considered as similarly precise indicators of the neuromuscular fatigue induced by acute resistance training protocols differing in level of effort when using the most typical intensity range in resistance training (70–90% 1RM).
▪ A given relative loss of MPV experienced within a set means that the level of induced fatigue is equivalent irrespective of the number of repetitions performed, at least in a range between 4 and 12 possible repetitions in the squat (SQ) and bench press (BP) strength training exercises.
▪ Capillary blood lactate concentration shows a linear relationship to the level of effort (LE) performed, in both SQ and BP exercises. Moreover, post-exercise lactate levels are highly correlated with the relative reductions in repetition velocity and CMJ height. Therefore, the blood lactate response to acute resistance exercise can be considered a good indicator of the level of effort performed.
▪ Ammonia concentration shows a curvilinear relationship to the level of effort (LE), increasing from resting levels when the number of performed repetitions within a set exceeds 50% of the number of possible repetitions against any load.
▪ Capillary blood lactate and ammonia levels show a curvilinear relationship to one another. Ammonia remains near resting levels until lactate exceeds ∼8 mmol/L in SQ and ∼6 mmol/L in BP, and then increases steadily as the level of effort increases.
▪ Ammonia increases from resting levels when the following relative reductions in mechanical variables do occur:
  ∼30% (SQ) and ∼35% (BP) reductions in MPV within a set
  ∼15% (SQ) and ∼20% (BP) reductions in MPV attained with the ∼1 m/s load
  ∼12% reduction in CMJ height
▪ Blood glucose level is independent of the level of effort performed.
▪ Serum testosterone concentration shows a linear relationship to the level of effort (LE) performed. Testosterone response is greater following SQ compared to BP exercise.
▪ The resistance exercise protocols that led to the greatest increases in serum testosterone levels were those performed with a level of effort of 12(12) and 10(10) in both exercises. These levels of effort were also the ones that induced the highest metabolic stress (lactate and ammonia post-exercise concentrations).
▪ All levels of effort led to increases in serum growth hormone (GH) levels. Increases in serum GH higher than 1000% were found for the following protocols: 12(12), 10(12), 8(12), 10(10), 8(8) in SQ; and 12(12), 10(12), 10(10), 6(6) in BP.
▪ The magnitude of GH response was clearly dependent on the muscle mass involved in the exercise. Thus, post-exercise serum GH levels were higher for SQ that BP with all levels of effort analyzed.
▪ Capillary blood lactate and ammonia levels showed a high correlation (r = 0,82-0,91) with serum GH levels following each of the 15 levels of effort analyzed.
▪ IGF-1 and C-Peptide hormones do not seem to be good indicators of the acute level of effort experienced during resistance exercise because both showed a very random response pattern in both exercises, following the 15 resistance training protocols.
▪ Serum cortisol levels showed a slight tendency to increase as the difference between the number of performed repetitions and the number of possible repetitions within a set was reduced, even though the differences between levels of effort were small and non-significant. Furthermore, cortisol levels were higher in SQ than in BP.
▪ It was observed a tendency towards increasing levels of serum insulin as the difference between the number of performed repetitions and the number of possible repetitions within a set was reduced, even though the differences were not statistically significant between levels of effort or exercises.
